# Response: Commentary: FSH receptor N680S genotype-guided choice of gonadotropin increases cumulative pregnancy and live birth rate after *in vitro* fertilization

**DOI:** 10.3389/fendo.2026.1832703

**Published:** 2026-04-24

**Authors:** Margareta Kitlinski, Yvonne Lundberg Giwercman

**Affiliations:** 1 Scanian University Hospital Malmö, Reproductive Medicine Center, Malmö, Sweden; 2 Lund university, department of Translational Medicine, Malmö, Sweden

**Keywords:** FSH, FSHR follicle-stimulating hormone receptor, IVF (*in vitro* fertilization, pregnancy, SNP

## *In vitro* experiments

The criticism regarding the absence of LH/hCG activity in our *in vitro* model reflects a misinterpretation of the study’s objective. No *in vitro* system—regardless of its biological complexity—can account for differences in pregnancy or live birth outcomes following ovarian stimulation with different FSH preparations. Our aim was not to compare granulosa or theca cell responses to Menopur versus Gonal F. Rather, it was to determine how two polymorphic variants of the FSHR respond to these hormones. Incorporating LH/hCG receptor signaling would have prevented isolation of FSHR-specific responses and thereby confounded the experimental design. It is noteworthy that in the very publication cited in support of this criticism (ref. 2), the authors themselves evaluated different FSH preparations in HEK293 cells transfected exclusively with FSHR. The methodological approach in that study mirrors our own in this respect.

We acknowledge that equivalent IU doses of Menopur and Gonal F correspond to approximately 30–50% higher molar amounts of Menopur. However, IU represents the clinically relevant dosing unit in controlled ovarian stimulation. Furthermore, this molar difference is unlikely to account for the approximately fourfold increase in cAMP production observed with stimulation of the SS FSHR variant across all tested concentrations. Importantly, Casarini and Simoni (ref. 2) reported a significantly higher EC_50_ for Menopur compared with Gonal F in HEK293 cells expressing FSHR alone, implying a lower FSHR-mediated response to Menopur when equivalent molecular doses are considered. Interpretation of their findings is additionally constrained by the absence of information regarding the specific N680S FSHR variant used for transfection.

Regarding statistical analysis, only two predefined comparisons were performed (10 IU and 90 IU). With p-values of 0.002 and 0.007, the results remain highly significant even after Bonferroni correction. While certain procedural details, such as final hormone concentrations in the buffer, were not explicitly specified, these omissions do not alter the validity of the findings.

## Clinical comparisons

With respect to the clinical component, the randomized study was registered as a two-center trial (ClinicalTrials.gov) but was completed at a single center due to insufficient recruitment at the second site during the COVID-19 pandemic. The retrospective inclusion of a non-genotyped control group is transparently described in the manuscript and comprised women who either declined participation or were not informed about the study. Assertions suggesting otherwise are unsupported.

Several additional points raised are not directly relevant to the stated objective of the study. Our purpose was to evaluate cumulative pregnancy and live birth outcomes in women not receiving FSH treatment selected according to their N680S FSHR genotype. Hence, in the prospective phase, hormone dosing was not genotype-guided, and genotypes were unknown at the time of treatment allocation. Standard dosing protocols were applied accordingly. The study was not designed to compare equivalent doses of different FSH preparations per se, but to determine whether genotype-guided selection improves clinical outcomes following IVF. A reference group was therefore included in which FSH preparation was chosen according to standard clinical practice. It is common practice to administer slightly higher IU doses of Menopur compared with recombinant FSH.

As specified in the manuscript, regression analyses were adjusted for female age and BMI as continuous variables, and a sensitivity analysis further adjusted for fertilization method (IVF vs. ICSI).

## Conclusion

Considering the clarifications provided above, the central postulate advanced by the authors cannot be considered sufficiently substantiated. Nonetheless, we welcome continued investigation in this area and agree that further studies will contribute valuable insight into genotype-guided ovarian stimulation.

